# Supplementary material for: Outcomes and complications among nonagenarians undergoing cardiac surgery: A scoping review
Source: PLoS One. 2025 Sep 8;20(9):e0331755. doi: 10.1371/journal.pone.0331755 (PMC12416686; doi:10.1371/journal.pone.0331755)
Supplement: S2 Table — (DOCX) [file pone.0331755.s002.docx]

**S2 Table.** Full search strategies for all electronic databases.

| **MEDLINE OVID 29 August 2023** | | |
| --- | --- | --- |
|  | Searches | Results |
| 1 | Heart surgery.mp | 21157 |
| 2 | Open heart surgery.mp | 9484 |
| 3 | Cardiac surgery.mp | 50303 |
| 4 | Coronary artery bypass graft.mp | 12445 |
| 5 | CABG.mp | 21301 |
| 6 | Aorta valve replacement.mp | 4 |
| 7 | Aortic valve replacement.mp | 27796 |
| 8 | 1 or 2 or 3 or 4 or 5 or 6 or 7 | 115175 |
| 9 | Nonagenarian.mp | 709 |
| 10 | Elderly.mp | 303962 |
| 11 | Very elderly.mp | 2642 |
| 12 | 9 or 10 or 11 | 304442 |
| 13 | Mortality.mp | 1395184 |
| 14 | 8 and 12 and 13 | 1765 |
| 15 | Limit 14 to (full text and abstracts and human and English language) | 357 |
| **EMBASE OVID 29 August 2023** | | |
|  | Searches | Results |
| 1 | Heart surgery.mp | 123320 |
| 2 | Open heart surgery.mp | 18332 |
| 3 | Cardiac surgery.mp | 80361 |
| 4 | Coronary artery bypass graft.mp | 93426 |
| 5 | CABG.mp | 39616 |
| 6 | Aorta valve replacement.mp | 18670 |
| 7 | Aortic valve replacement.mp | 41998 |
| 8 | 1 or 2 or 3 or 4 or 5 or 6 or 7 | 2276946 |
| 9 | Nonagenarian.mp | 1115 |
| 10 | Elderly.mp | 713526 |
| 11 | Very elderly.mp | 291270 |
| 12 | 9 or 10 or 11 | 713796 |
| 13 | Mortality.mp | 1985132 |
| 14 | 8 and 12 and 13 | 6727 |
| 15 | Limit 14 to (full text and abstracts and human and English language) | 1464 |
| **Cochrane Library (Wiley Online Library) 29 August 2023** | | |
|  | Searches | Results |
| 1 | (heart surgery OR open heart surgery OR cardiac surgery OR coronary artery bypass graft OR aorta valve replacement OR aortic valve replacement):ti,ab,kw | 44080 |
| 2 | (nonagenarian OR very elderly OR elderly):ti,ab,kw | 56551 |
| 3 | (mortality):ti,ab,kw | 109379 |
| 5 | 1 and 2 and 3 | 652 |
| 6 | Limit 5 to (Cochrane reviews and protocols) | 5 |
