# Supplementary material for: Outcomes and complications among nonagenarians undergoing cardiac surgery: A scoping review
Source: PLoS One. 2025 Sep 8;20(9):e0331755. doi: 10.1371/journal.pone.0331755 (PMC12416686; doi:10.1371/journal.pone.0331755)
Supplement: S3 Table — (DOCX) [file pone.0331755.s003.docx]

**S3 Table.** Cardiopulmonary bypass and aortic cross-clamp: times reported by the publications included in the review.

| Authors | Cardiopulmonary bypass (CPB)  time (minutes) | Aortic cross clamp  (ACC)  time (minutes) |
| --- | --- | --- |
| Assmann et al. (2013) [32] | 131 | 88 |
| Bacchetta et al. (2003) [21] | 102 | 60 |
| Blanche et al. (1997) [19] | 126 | 91 |
| Easo et al. (2011) [31] | 152 | 58.5 |
| Edwards et al. (2003) [23] | 95 | Not reported |
| Elsisy et al. (2021) [42] | 75 | 53 |
| George et al. (2016) [37] | 109 | 79.1 |
| Guilfoyle et al. (2008) [27] | 100 | 65 |
| Levy Praschker et al. (2006) [24] | 70.1 | 50.7 |
| Miller et al. (1999) [20] | 90 | 50 |
| Murashita et al. (2014) [35] | 60 | 45 |
| Samuels et al. (1996) [18] | 102 | 64 |
| Tsai et al. (1994) [17] | 138 | Not reported |
| Ullery et al. (2008) [28] | 94.4 | 52.7 |
| Weinberg et al. (2022) [44] | 121 | 95 |
