# Supplementary material for: Outcomes and complications among nonagenarians undergoing cardiac surgery: A scoping review
Source: PLoS One. 2025 Sep 8;20(9):e0331755. doi: 10.1371/journal.pone.0331755 (PMC12416686; doi:10.1371/journal.pone.0331755)
Supplement: S4 Table — (DOCX) [file pone.0331755.s004.docx]

**S4 Table.** Overall complication rates listed by type.

|  | Incidence of complications | | | | |
| --- | --- | --- | --- | --- | --- |
| Type of complication | **Minimum** | **25^th^ quartile** | **Median** | **75^th^ quartile** | **Maximum** |
| Any complication | 33.3 | 56 | 71 | 76 | 100 |
| Pulmonary (pneumonia, respiratory failure or prolonged mechanical ventilation) | 3.3 | 11.6 | 19.4 | 29.2 | 37 |
| Return to theatre for bleeding | 2.8 | 3.7 | 8.6 | 12.3 | 36.7 |
| Stroke | 0.8 | 3 | 4.9 | 7.9 | 18 |
| Low cardiac output syndrome and postoperative myocardial injury | 4.4 | 6.5 | 20 | 34 | 63 |
| Postoperative delirium | 5.2 | 7.5 | 17.6 | 28.6 | 39 |
| Gastro-intestinal bleeding | 1.7 | 3.7 | 5.3 | 7.2 | 7.5 |
| Infection (sepsis or deep sternal wound infection) | 0.4 | 4.6 | 8.7 | 13.3 | 18 |
| Arrhythmia (atrial fibrillation or flutter, ventricular fibrillation or tachycardia) | 17.6 | 26.7 | 45 | 55 | 86 |
| Permanent pacemaker insertion | 5.6 | 6.3 | 10.1 | 13.5 | 16 |
| Acute kidney injury | 5.6 | 9.5 | 13.8 | 21.2 | 78 |
