# Supplementary material for: Outcomes and complications among nonagenarians undergoing cardiac surgery: A scoping review
Source: PLoS One. 2025 Sep 8;20(9):e0331755. doi: 10.1371/journal.pone.0331755 (PMC12416686; doi:10.1371/journal.pone.0331755)
Supplement: S5 Table — (DOCX) [file pone.0331755.s005.docx]

**S5 Table.** Length of hospital and intensive care stay.

| Author | Length of hospital Stay  (days) | Length of ICU Stay  (days) |
| --- | --- | --- |
| Assman et al. (2013) [32] | Not reported | 3.2 |
| Baccheta et al. (2003) [21] | 19.8 | 12 |
| Blanche et al. (1997) [19] | Not reported | 18.6 |
| Bridges et al. (2003) [22] | 8 | Not reported |
| Davis et al. (2014) [34] | 11 | 5.2 |
| Easo et al. (2011) [31] | 17.6 | 7.05 |
| Edwards et al. (2003) [23] | 18 | Not reported |
| Elgendy et al. (2019) [41] | 12.5 | Not reported |
| Elsisy et al. (2021) [42] | 7 | Not reported |
| George et al. (2016) [37] | 16.5 | 10.9 |
| Gulifoyle et al. (2008) [27] | Not reported | 2 |
| Hovanesyan et al. (2007) [26] | 12.2 | 3.4 |
| Mack et al. (2015) [36] | 10.35 | Not reported |
| Miller et al. (1999) [20] | 28 | Not reported |
| Murashita et al. (2014) [35] | 8 | Not reported |
| Samuels et al. (1996) [18] | 25.7 | Not reported |
| Speziale et al. (2010) [30] | Not reported | 10.2 |
| Weinberg et al. (2022) [44] | 11.6 | Not reported |
| Zack et al. (2017) [40] | 14.1 | Not reported |
