# Supplementary material for: Outcomes and complications among nonagenarians undergoing cardiac surgery: A scoping review
Source: PLoS One. 2025 Sep 8;20(9):e0331755. doi: 10.1371/journal.pone.0331755 (PMC12416686; doi:10.1371/journal.pone.0331755)
Supplement: Attachment — Submitted filename: RESPONSE TO REVIEWERS_31March2025.pdf [file pone.0331755.s008.pdf]

Professor Marcelo Arruda Nakazone, M.D., Ph.D.
Academic Editor
PLoS ONE

Ref: Resubmission of PONE-D-24-26035

Title: Outcomes and complications in nonagenarians undergoing cardiac surgery: a scoping review

Dear Dr. Nakazone:

My coauthors and I would like to thank you and the expert reviewers for taking the time to provide a very constructive and thoughtful review of our manuscript.

As requested, we have included the following items with our resubmission:

1. A point-by-point response letter (to follow) that addresses each issue raised by the Academic Editor and the Reviewers. I have uploaded this letter as a separate file labelled "Response to Reviewers."
2. A marked-up copy of the manuscript that highlights changes made to the original version. This has been uploaded as a separate file labelled "Revised Manuscript with Tracked Changes."
3. An unmarked version of our revised paper without tracked changes. This has also been uploaded as a separate file labelled "Manuscript."

Journal Requirements

Question 1: Please ensure that your manuscript meets PLOS ONE's style requirements, including those for file naming. The PLOS ONE style templates can be found at https://journals.plos.org/plosone/s/file?id=wjVg/PLOSOne_formatting_sample_main_body.pdf and https://journals.plos.org/plosone/s/file?id=ba62/PLOSOne_formatting_sample_title_authors_affiliations.pdf

Authors' response to Q1: Thank you for this important comment. The text has been edited and formatted and is now consistent with the style guidelines provided above.

Question 2. We noticed you have some minor occurrence of overlapping text with the following previous publication(s), which needs to be addressed: https://bmjopen.bmj.com/content/13/7/e072293.full. In your revision ensure you cite all your sources (including your own works), and quote or rephrase any duplicated text outside the methods section. Further consideration is dependent on these concerns being addressed.

Authors' response to Q2: The manuscript noted at this website (https://bmjopen.bmj.com/content/13/7/e072293.full), entitled "Outcomes and complications of nonagenarians undergoing cardiac surgery: a scoping review protocol" (Ludski et al.) is our earlier publication of the protocol used to guide the study described here. We have now cited this in the text) and also included this as reference #15. As noted in the text, we published the protocol to ensure more effective dissemination, public accessibility, and transparency. Of necessity, we repeated some of the text from this publication (as noted above, minor) to provide clarity throughout.

We are pleased to inform you that we have carefully rephrased any duplicated text in our manuscript titled "https://bmjopen.bmj.com/content/13/7/e072293.full; "Outcomes and complications of

nonagenarians undergoing cardiac surgery: a scoping review protocol" (Ludski et al.) to ensure originality and clarity. Additionally, The University of Melbourne has conducted a thorough review using iThenticate to verify that the content does not overlap with our prior publications. The similarity index for the manuscript is below 10%, with all flagged matches appropriately attributed to cited sources. We are happy to provide this information if requested. We are confident these revisions align with the journal's ethical standards and formatting guidelines.

Should the editorial team or reviewers recommend further adjustments, we are fully committed to addressing them promptly.

Question 3. We note that there is identifying data in the Supporting Information file <Supplementary Table 2 .docx. Due to the inclusion of these potentially identifying data, we have removed this file from your file inventory.

Authors' response to Q3: Thank you for notifying us of this oversight.

Reviewer ONE

We thank Reviewer One for their expert insights and time spent reviewing our manuscript. We have provided a detailed comment to each of the reviewer's questions, as outlined below.

1. **Reviewer 1, comment 1:** This systematic review on outcomes and complications in nonagenarians undergoing cardiac surgery addresses a relevant and timely topic. It provides important insights into mortality rates, complications, and hospital stays in this age group. However, there are several areas where the manuscript could be improved.

Strengths: The topic is highly pertinent given the aging population and increasing number of elderly surgical candidates. The literature search is thorough, covering multiple countries and procedures. PRISMA guidelines were followed.

Authors' response to R1 comment 1: Thank you for these positive comments.

Minor Concerns

2. **Reviewer 1, comment 2:** The introduction could be streamlined to focus more on the specific gaps in the literature that this review addresses.

Authors' response to R1 comment 2: Done. The Introduction has been streamlined and now focuses primarily on the goals of the scoping review. Thank you for this constructive comment.

3. **Reviewer 1, comment 3:** The rationale for excluding non-English articles and certain cardiac procedures could be more clearly explained, as this may limit the generalizability of findings.

Authors' response to R1 comment 3: We have expanded our rationale for excluding articles not written in English (note that we did include articles with English-language abstracts and had the full papers translated if they fulfilled the study criteria). Interestingly, a 2021 study published by Dobrescu and colleagues in the *Journal of Clinical Epidemiology* reported that restricting selections to English-

language publications appeared to have little impact on the effect estimates and conclusions of systematic reviews. This information has been included together with a new reference [16].

Also, we thought that rather than explaining the cardiac procedures (which would add quite a bit of extra text to the manuscript), the reader might refer to the original studies (references [17-44]) for further details. However, we have expanded the list of cardiac procedures as outlined in the revised manuscript.

4. **Reviewer 1, comment 4:** The statistical methods are not adequately described. More detail on data synthesis and analysis, particularly handling of heterogeneity (if any) among studies, is needed. The statistical methods used for data synthesis may be described in more detail. While the manuscript mentions the use of descriptive statistics, there is a lack of detail on how these were calculated and whether any meta-analytic techniques were applied. The authors could consider providing a more robust analysis or explaining why certain methods (e.g. meta-analysis) were not feasible.

Authors' response to R1 comment 4: Thank you for this constructive comment.

The primary aim of our scoping review was to map the available evidence rather than generate a pooled effect estimate. Differences in outcomes or patient characteristics across studies were discussed narratively.

There were several reasons why meta-analytic techniques could not be used in our scoping review. All our included studies typically ranged from very small case series to large observational studies. There were no randomised controlled trials included. Of the 28 studies we included, 20 (71.4%) had less than 50 patients included. There were 12 studies (42.9%) with less than 25 patients. This methodological diversity makes it inappropriate to combine effect sizes statistically. More importantly, many of the outcome measure were variable. Studies reported outcomes such as complications using different definitions. Timeframes, measurement tools also varied preventing direct statistical comparison. The included papers comprised various types of cardiac surgeries, from coronary bypass to complex major aortic interventions, making a single pooled estimate meaningless.

Our data synthesis and analysis were conducted systematically to ensure rigor and reliability. All continuous data were tested for normality using the Kolmogorov–Smirnov test and/or visual inspection of the Q-Q plot. For data that followed a normal distribution, we reported means and standard deviations, while for non-normally distributed data, we utilized medians and interquartile ranges [IQRs], and ranges (minimum-to-maximum values) to summarize the findings. Differences in categorical variables were assessed using either the Chi-square test or Fisher's exact test, depending on the sample size and distribution of the data.

In studies where heterogeneity was evident, a narrative synthesis approach was planned to enhance understanding of patient-reported outcomes. This approach involved identifying research gaps through a comparative analysis of study designs and participant characteristics. Additionally, where feasible, thematic analysis was undertaken to explore references to quality-of-life outcomes among nonagenarians. The thematic analysis process included familiarization with the data, generation of codes to capture key concepts related to outcomes, complications, and management strategies. This methodological flexibility allows for the nuanced exploration of the complexity present in the literature while maintaining adherence to a coherent analytical framework. This framework facilitates the identification of knowledge gaps and the recognition of emerging trends within the field.

This has been updated in the resubmission manuscript.

5. **Reviewer 1, comment 5:** The discussion should more critically evaluate the findings, particularly the high complication rates, and offer a more balanced view on surgical risks and benefits. For example, the implications of the high complication rates could be explored in greater depth, particularly concerning how these might influence surgical decision-making in nonagenarians. The manuscript suggests that age should not be a disqualifying factor for surgery, which is an important point. However, this statement would benefit from a discussion of the balance between risks and benefits, considering patient preferences and quality of life post-surgery.

Authors' response to R1 comment 5: Thank you for this constructive comment.

We agree that the elevated complication rates observed in nonagenarians undergoing cardiac surgery have significant implications for surgical decision-making in this cohort. Although advanced age alone should not preclude surgical intervention, the heightened risk of complications and mortality warrants careful consideration. Extant literature reports postoperative complication rates reaching 60% in nonagenarians undergoing major surgery, with a one-year mortality rate of approximately 20% following cardiac procedures.

We also agree that these substantial risks necessitate comprehensive discussions with patients and their families to facilitate informed decision-making. Surgeons must carefully balance the potential benefits of surgery against the associated risks, while considering patient preferences and anticipated postoperative quality of life. In assessing surgical candidacy, factors such as frailty, comorbidities, and functional status should be evaluated in conjunction with age. Moreover, the urgency of the procedure is a critical consideration, as elective surgeries generally yield more favorable outcomes compared to urgent or emergent cases. Ultimately, the decision to proceed with cardiac surgery in nonagenarians should be individualized, taking into account not only survival prospects but also the potential for improved functional status and quality of life.

Interestingly, among our major findings, we noted that we could find almost no published information that directly addresses this extremely important question. Although we collected some information on complication rates, which, intriguingly, appear to be somewhat lower than those cited for noncardiac surgery in this population, none of the studies reported patient-centred outcome measures. For example, we could find little to no information on patient days alive and out of the hospital, patient-reported outcome measures, or patient-reported experience measures.

We have taken your suggestion and added additional information and emphasis on this point to the statements regarding age and potential complication rates.

This has been updated in the discussion section under the section titled "Complications".

6. **Reviewer 1, comment 6:** The manuscript identifies a lack of patient-reported outcomes but could suggest how future research might address this gap.

Authors' response to R1 comment 6: Done. In the section addressing Future Directions, we have included a more extensive discussion of tools used to measure these outcomes that might be validated in this very elderly cohort (new references [71,72]).

7. **Reviewer 1, comment 7:** There are a few typographical errors and grammatical issues throughout the manuscript. A thorough proofreading would be beneficial. For example, the sentence "From the initial 1015 articles searched, we included twenty-eight studies from eight countries were included" is redundant and should be revised for clarity.

Authors' response to R1 comment 7: The original manuscript has undergone thorough proofreading. All typographical errors and grammatical issues have been addressed, and additional changes have been made to improve clarity and readability throughout.

8. **Reviewer 1, comment 8:** Ethical Considerations: A discussion of the ethical implications of surgery in nonagenarians, particularly around informed consent, would be valuable.

Authors' response to R1 comment 8: Done. We have highlighted this specific concern in the Discussion section (with new references [45,46]).

Reviewer TWO

We thank Reviewer Two for their expert insights and time spent reviewing our manuscript. We have provided a detailed comment to each of the reviewer's questions, as outlined below.

1. **Reviewer 2, Comment 1:** The authors have to make a choice between a systematic and a scoping review that they seem to assimilate (even in title) but which are two different type of studies with different aim and methodology. See Munn, Z., Peters, M.D.J., Stern, C. et al. Systematic review or scoping review? Guidance for authors when choosing between a systematic or scoping review approach. BMC Med Res Methodol 18, 143 (2018). https://doi.org/10.1186/s12874-018-0611-x.

Authors' response to R2 comment 1: Thank you for this important question. We appreciate the opportunity to clarify our rationale for conducting a scoping review rather than a systematic review.

The primary reason for choosing a scoping review methodology is the current state of the literature in this area. Given the relatively sparse and heterogeneous body of research regarding the outcomes of cardiac surgery in nonagenarians, the use of the scoping review format permitted us to generate a comprehensive map of all available evidence, identify key themes, and highlight gaps in the literature. Unlike a systematic review, which focuses on synthesizing high-quality studies to answer a specific research question, a scoping review provides a broader overview of the extent, range, and nature of the existing research.

Additionally, a scoping review is particularly suited for an emerging or complex area such as this, where variations in study design, outcome measures, and patient cohorts make direct comparison difficult. By capturing a wide spectrum of evidence, including retrospective cohort studies, case series, and observational studies, we can better understand the breadth of available data and assess whether a more targeted systematic review would be feasible in the future. Furthermore, our approach aligns with best practices for evidence synthesis in areas with limited randomized controlled trials or standardized reporting. This methodology enables us to categorize different study types, methodologies, and reported outcomes without the stringent inclusion criteria that a systematic review would necessitate.

In summary, our decision to conduct a scoping review stems from the need to systematically explore and describe the literature landscape in this field. We believe this approach has provided valuable insights into the current knowledge base that will inform future research directions and ultimately support improved clinical decision-making for this vulnerable patient population.

To clarify this point, we have changed the title to "*Outcomes and complications in nonagenarians undergoing cardiac surgery: a scoping review*" and adjusted the text accordingly.

2. **Reviewer 2, Comment 2:** One strength of the study is the extended review of literature and the rigorous methodology deployed to perform the analysis since out of 1015 articles searches (with no restriction on study design or date of publication), the authors included 28 studies from 8 countries (most of them issued from US centers) and totalizing a sample size of 6411 patients.
   However, the majority of those papers are older than 10 years and certainly do not reflect medical practice in the current era where endovascular procedures has become the main treatment carrier in nonagenarian patients in all domains of cardiovascular diseases including structural valve pathology. Those papers totalize 17% of the overall population but it is not specified how did the authors ensure that patients included in the more recent studies of Khalid, Zack and Elgendy, did not already appear in previous published studies from US. Therefore, in the current review, the probability for one patient to have been reported at least twice is significant.

Authors' response to R2 comment 2: Thank you for this very important question. We acknowledge that patients included in our review from The Society of Thoracic Surgeons National Database [23] may overlap with patients in 9 other studies included in this review. This potential crossover is an important consideration in interpreting our findings. studies. Even when these studies are excluded from the analysis, our finding are unchanged. This further highlights the need for future research to more precisely quantify the extent of patient overlap in multi-center cardiac surgery studies.

Finally, we acknowledge that a significant proportion of the papers included in our analysis are older than 10 years, which may not fully reflect current medical practices, particularly given the increasing prominence of endovascular procedures in treating nonagenarian patients across various cardiovascular domains, including structural valve pathology. However, these studies provide historical context and insight into the evolution of cardiac surgery in nonagenarians, highlighting advancements and shifts in treatment paradigms over time and offer a broader perspective on long-term outcomes and complications, which are crucial for understanding the durability and effectiveness of interventions.

This has been added to the revised manuscript under the "Strengths and limitations" section.

3. **Reviewer 2, Comment 3:** Since a scoping review focuses on the importance of one specified subject in the literature, it is expected that the outcome would relate on one bibliometric index such as the annual incidence of papers related to the subject under study. Instead, the authors have used traditional clinical outcomes such as in-hospital mortality, morbidity and length of stay. It even appears that they may have discarded some relevant papers which did not included those clinical outcomes. This is deeply confounding. If they authors aim to perform a systematic review or a metanalysis of the clinical outcomes of cardiac surgery in nonagenarian patients, then the title must be changed as well as the paper has to be deeply revised accordingly.

Authors' response to R2 comment 3: We thank the reviewer for the opportunity to emphasize our goals in this manuscript. As discussed above in response to the comment 1 of Reviewer #1, the original title and some of the text misstated our true intent. As originally described in our protocol publication (reference [15]), we intended to perform a *scoping review* of the literature, defined as a method that might be used to provide a broader overview of the extent, range, and nature of the existing research. Within this format, our goal was to assess whether a deficiency exists in the published literature and potentially identify knowledge gaps to guide future efforts designed to improve our understanding of nonagenarians undergoing cardiac surgery.

To the end, we performed an extensive search and identified only 28 papers **that met our inclusion criteria** (see Table 1 and also in our published protocol [15]). As noted in the text, we followed the guidelines provided by the PRISMA Extension for Scoping Review Protocol in preparing our findings. We are unaware of any relevant manuscripts that fit these criteria that were discarded. Among our most important conclusions, we note the near-complete absence of any findings that address post-operative quality of life issues in this fragile patient cohort. We identify this as a critical gap in the literature and our current knowledge and point to the need for future research to improve our critical understanding of this subject.

4. **Reviewer 2, comment 4:** The discussion and interpretation reflect more the author's belief than the result that they present. Based on those results I would rather assume that the relatively seldom number of papers found in the literature concerning cardiac surgery in nonagenarian patients is a marker of the reluctance to propose chest opening and CPB to frail patients with limited life expectancy. Most of them would probably deny it anyway unless they have the feeling of an imminent death or, most likely, even though. If the well-looking conclusion (not to deny cardiac surgery because of patient's age only) seems quite reasonable, it is hardly supported by the presented results for a 10% mortality rate for elective procedures is not deemed acceptable in today standards of cardiac surgery.

Authors' response to R2 comment 4: We appreciate your insightful comment, which has prompted robust discussions amongst our authors. We agree with your valuable observation and have now adjusted the concluding text to reflect this reasonable interpretation of our findings. Specifically, we have revised the relevant section (see lines 380–382) to better align with your suggestions. Thank you for your contribution to enhancing the clarity and impact of our manuscript.

In the concluding paragraph we now state "*This scoping review highlights that while age alone should not be a barrier to cardiac surgery for nonagenarians, the procedure is associated with significantly increased morbidity and a relatively high mortality rate. Notably, one in ten patients in this age die, and two-thirds of patients succumb to complications within 30 days post-surgery. Most patients experience extended intensive care stays. Despite these challenges, most patients are ultimately discharged from the hospital. A critical gap in the literature is the scarcity of data on quality-of-life measures and patient-reported outcomes, which are essential for informed clinical discussions and consent. Incorporating these factors into decision-making processes is crucial to ensure that care aligns with patients' values and preferences, particularly in this vulnerable population. Future research should prioritize the collection and analysis of these outcomes to enhance the quality and relevance of care for nonagenarians undergoing cardiac surgery.*

Our review highlights significant gaps in our collective knowledge base and specific areas in need of further research, including standardised methods for reporting complications, patient-centred outcomes, functional recovery metrics, and long-term mortality data. This will provide us with a better understanding of patient selection criteria, as well as the perioperative and postoperative experiences of nonagenarians undergoing cardiac surgery.

We would be very happy to make further adjustments if needed.

Reviewer THREE

We thank Reviewer Three for their expert insights and time spent reviewing our manuscript. We have provided a detailed comment to each of the reviewer's questions, as outlined below.

1. **Reviewer 3, comment 1:** Thee authors present a scoping review of the literature to look at the outcomes of cardiac surgery in nonagenarians. They have published their protocol and registered the review. The standard of English is generally good although there are scattered typos and grammatical errors that require a thorough check through the paper (e.g. line 138, redundant "The"s). The following comments are designed to help the authors address some conflicts between protocol and paper.

Authors' response to R3 comment 1: Thank you for these positive comments. As noted above in response to Comment 7 of Reviewer #1, the original manuscript has undergone thorough proofreading. All typographical errors and grammatical issues have been addressed, and additional changes have been made to improve clarity and readability throughout.

2. **Reviewer 3, comment 2:** There is differing definitions of the Population. In some sections, it is >90y, in others 90-99. Clarify which - and in cases where papers presented mixed data, how this was resolved

Authors' response to R3 comment 2: Thank you for your feedback regarding the consistency of our population definitions. We appreciate your thorough review and the opportunity to address this important aspect of our study. In response to your comment, we assure you that have carefully reviewed all definitions of the population throughout the manuscript.

As a result of this comprehensive review, we have ensured that the population definition is consistent across all sections, including the introduction, methods, results, and discussion. Second, any discrepancies or ambiguities in the population description have been addressed and clarified. Finally, we have standardized the terminology used to refer to our study population to avoid any potential confusion.

3. **Reviewer 3, comment 3:** The paper suggests it will include grey literature, but largely excludes by design the large national registries that might describe this information (e.g., although outside the search dates, the SWEDEHEART registry of nonagenarians published recently)

Authors' response to R3 comment 3: We are hoping to expand the important findings that emerged from this scoping review with a "deep dive" into one or more large national registries. We have included this point in the section on Future Research (new reference [74]).
Of note in the published SWEDEHEART registry paper (https://doi.org/10.1093/ehjqcco/qcab039), the highest age group reported was patients aged 75-79 years. This registry does not focus specifically on patients who have undergone open cardiac surgery but rather focuses on patients who have had an acute myocardial infarction, from hospital care to secondary prevention. The aim of the SWEDEHEART-CR registry is to provide continuous information on secondary prevention and cardiac rehabilitation performance to support the audit and development of evidence-based practice.

4. **Reviewer 3, comment 4:** Line 148 - please clarify, were full papers translated?

Authors' response to R3 comment 4: If the papers in question were published with English-language abstracts and otherwise fulfilled the eligibility criteria, the full manuscript was translated into English and subjected to a comprehensive evaluation.

5. **Reviewer 3, comment 5:** Line 151 - please clarify, this sentence is ambiguous

Authors' response to R3 comment 5: As noted in our response to Comment 3 of Reviewer #1, the text of this section has been changed, and a new reference has been added. It now reads as follows: **"…***this decision aligned with the exploratory intent of our scoping review, which was primarily aimed at mapping the extent of the literature available for immediate review. Interestingly, results from a recent review revealed that restricting selections to English-language publications only has little impact on effect estimates and relevant conclusions [16]."*

6. **Reviewer 3, comment 6:** Line 156 - centenarians included here

Authors' response to R3 comment 6: Thank you for pointing out this error. This has now been corrected. The sentence now reads "*As the primary aim of this scoping review was to provide a wide overview of the field, our eligibility criteria included nonagenarians who had undergone a wide variety of cardiac surgical procedures"*

7. **Reviewer 3, comment 7:** Line 159 - lots of procedures not in the search criteria

Authors' response to R3 comment 7: The review methodically addresses interventions according to established classification systems, including the OPCS coding framework and Australian Classification of Health Interventions. This approach ensures comprehensive coverage of all cardiac surgical domains, which consistently demonstrate similar distribution patterns across international registries with CABG, valve procedures, and congenital surgeries comprising the predominant operations. We have updated the inclusion criteria to include heart transplantation, open ventricular assist device implantation, and open left ventricular aneurysmectomy.

We initially excluded heart transplantation, as the International Society for Heart and Lung Transplantation (ISHLT) revised guidelines to consider heart transplantation for patients in their seventies. Analysis of the United Network for Organ Sharing (UNOS) database (1987–2014) reveals that 1.4% of 50,432 heart transplant recipients were ≥70 years old, with a maximum age of 79. Whilst the number of heart transplants in patients ≥65 years is increasing, to our knowledge this procedure has never been undertaken in a nonagenarian.

Reference: Bortolussi G, et al. Identifying cardiac surgery operations in hospital episode statistics administrative database, with an OPCS-based classification of procedures, validated against clinical data. BMJ Open. 2019 Mar 23;9(3):e023316. doi: 10.1136/bmjopen-2018-023316.

8. **Reviewer 3, comment 8:** Information sources are not outlined as per PRISMA guidelines - how were authors contacted etc.

Authors' response to R3 comment 8: The decision to forgo author contact in our scoping review aligns with its fundamental purpose of mapping the breadth and nature of existing evidence rather than synthesizing quantitative outcomes or assessing methodological rigor. Scoping reviews, by definition, prioritize identifying knowledge domains, conceptual boundaries, and research gaps across heterogeneous literature—objectives that differ fundamentally from systematic reviews requiring granular data extraction or risk-of-bias evaluations. While systematic reviews often rely on author correspondence to resolve ambiguities in reported outcomes or methodologies for meta-analytic purposes, such practices prove unnecessary when the primary aim involves cataloguing study characteristics, intervention types, and theoretical frameworks.

Our methodology adheres to established scoping review frameworks from the Joanna Briggs Institute (JBI) and PRISMA-ScR guidelines, which emphasize comprehensive database searches and descriptive analysis over investigator engagement. These protocols explicitly distinguish scoping reviews from systematic reviews by their exclusion of quality appraisal processes and data

synthesis—the very contexts where author contact becomes critical. Furthermore, empirical evidence demonstrates that 72% of scoping reviews omit author outreach without compromising their ability to characterize research landscapes, as missing data points or unclear reporting in individual studies do not obstruct thematic mapping objectives. By focusing on publicly available information, we maintain methodological consistency with scoping review best practices while avoiding the 22–68% nonresponse rates and time delays associated with author correspondence, ensuring efficient resource allocation toward our review's core exploratory aims.

9. **Reviewer 3, comment 9:** Line 193 - not clear how two reviewers can agree on four and five studies and end with nine studies? Surely agreement would mean including just four studies (and one that was not independently agreed on).

Authors' response to R3 comment 9: Thank you for pointing this out. This has now been corrected and clarified in the revised manuscript.

10. **Reviewer 3, comment 10:** Line 239 - PPI is possible even in reviews

Authors' response to R3 comment 101: PPI (patient and public involvement) is certainly possible in reviews, but it was not a part of this study. The text has been adjusted accordingly.

Patient and public involvement was not directly incorporated into the methodology of this scoping review due to its specific focus on mapping the existing literature landscape. The primary objective was to comprehensively catalogue and analyze the current body of evidence, including any studies that reported Patient-Reported Outcome Measures (PROMs) or patient-centered quality-of-life outcomes. While PPI is invaluable in many research contexts, particularly in primary studies and in shaping research priorities, this review's scope was deliberately constrained to synthesizing published data. The inclusion of studies reporting PROMs and patient-centered outcomes ensured that patient perspectives, as captured in the existing literature, were represented in the review's findings. This approach allowed for a systematic and objective assessment of the current state of evidence, providing a foundation for future research where direct patient and public involvement could be more appropriately integrated.

11. **Reviewer 3, comment 11:** Ref 23 - this large study from the STS database likely crosses over some of the patients from the other studies. How did the authors account for this?

Authors' response to R3 comment 11: Thank you for raising this important question. It is indeed possible that some patients from The Society of Thoracic Surgeons National Database may overlap with patients included in other studies analyzed in our review. This potential crossover is an important consideration in interpreting our findings. We have accounted for this possibility in several ways: firstly, our data extraction process carefully documented the source and time frame of each study to identify potential overlaps.as seen in Table 2, we have outlined the dates of the searches and well as the countries that each study was conducted. We have carefully reviewed the inclusion criteria of the 28 studies we have included. The reviewer is correct in that it is possible that patients from 9 studies. Even when these studies are excluded from the analysis, our finding are unchanged. In our analysis, we have been cautious in interpreting results from studies that may have overlapping patient populations.

We have now explicitly addressed this issue in the limitations section of our paper. Specifically, we have, acknowledged the potential for patient crossover between large database studies, discussed the implications of this crossover on the interpretation of our findings, and highlighted the need for future

research to more precisely quantify the extent of patient overlap in multi-center cardiac surgery studies.

By addressing this limitation transparently, we aim to provide readers with a more nuanced understanding of our results and their applicability to clinical practice.

12. **Reviewer 3, comment 12:** Line 274 - Figure 2 does not show operations, but this information would be useful and is not shown elsewhere

Authors' response to R3 comment 12: Thank you for this important comment. We have now included a revised Figure 4 (see below)that outlines the types of surgeries included in the labelling. The Table legend further expands on whether these are CABG, aortic surgery, aortic valve surgery, or mixed surgeries.

Fig 4. Mortality stratified by each study, together with the types of cardiac surgical procedures performed. SAVR: surgical aortic valve replacement; CABG: coronary artery bypass; Type AoD: type A aortic dissection repair. Mixed refers to aortic, mitral or tricuspid valve repair or replacement (or a combination of these surgeries)

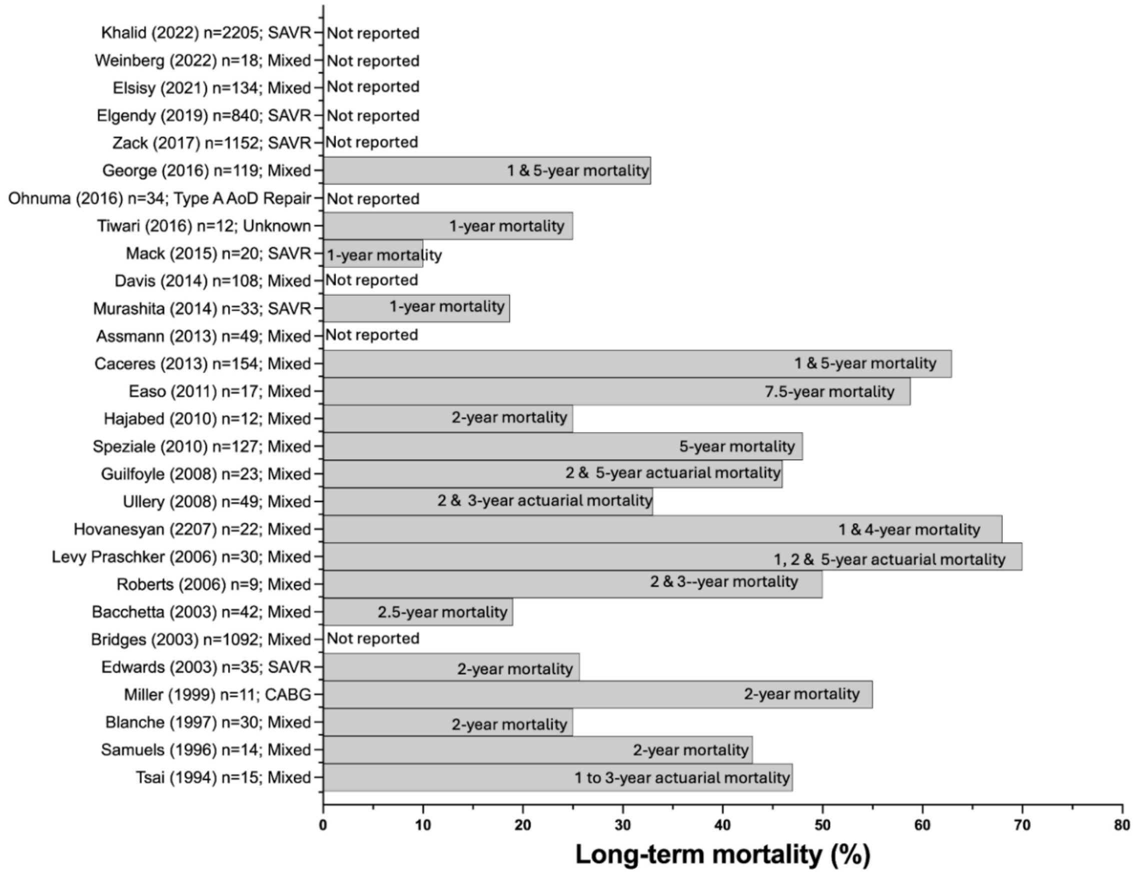

13. **Reviewer 3, comment 13:** 278 - was additional detail provided in none of the 28 studies? This seems a surprise

Authors' response to R3 comment 13: We agree with the reviewer, however a detailed review of each study found that there was a dearth of patient reported outcomes stated.

14. **Reviewer 3, comment 14:** 289 - was risk stratification also provided e.g. EuroSCORE or STS score?

Authors' response to R3 comment 14: Thank you for highlighting this important point. We concur with your assessment of the significance of this information and have taken steps to address it comprehensively. In response, we have conducted a thorough review of all included papers to determine the presence and type of risk scoring systems reported. The results of this analysis have been incorporated into the Results section of our manuscript.

Our findings can be summarized as follows "*Twenty-one studies (77.8%) reported some form of a risk stratification score. Among these, 19 studies (70.4% of total) utilized the New York Heart Association (NYHA) functional classification, four studies (14.8% of total) employed EuroScores, three studies (11.1% of total) used the American Society of Thoracic Surgeons (STS) score, and one study (3.7% of total) applied the Charlson Comorbidity Index.*"

This has been included in the updated manuscript.

15. **Reviewer 3, comment 15:** Fig 2 - this figure is complex. Would a bubble plot help to show the same data in a more condensed form, with the size of the bubble indicating the size of the study, x being time and y being survival?

Authors' response to R3 comment 15: Thank you for this excellent suggestion. We have updated Figure 2 to include a bubble plot. illustrating in-hospital or 30-day mortality rates (%) across studies published between 1992 and 2024. Each bubble represents a study, with bubble size corresponding to the number of patients included in the study (as indicated by the color gradient scale). Larger bubbles and warmer colors denote studies with higher patient counts. The x-axis represents the year of publication, while the y-axis shows in-hospital or 30-day mortality rates.

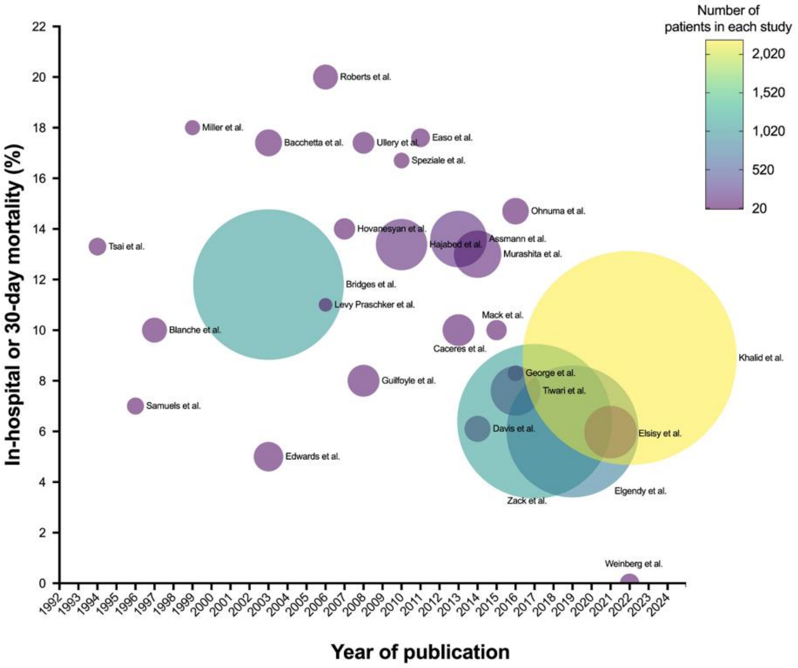

16. **Reviewer 3, comment 16:** Fig 3 - this summary figure was much more helpful and easily to interpret

Authors' response to R3 comment 16: Thank you, we are happy to hear this.

17. **Reviewer 3, comment 17:** 303-313 - complication rates are very difficult to assess when presented in isolation; some of these are common in any age group, some may be likely in certain procedure groups. A more descriptive narrative of the findings would help the reader.

Authors' response to R3 comment 17: We appreciate this suggestion. In response, we have added some additional information on the outcomes of open-heart procedures reported in the general (routinely younger) population. For example, a 2023 study published in JAMA Surgery (Gaudino et al., new reference [48]) that included more than 1.2 million patients reported that the complication rate of coronary artery bypass grafting was only 15% among those 59 – 73 years of age. Similarly, Bis et al. published a study in JTCVS (new reference [49]) on postoperative conduction disturbances that included 15,000 patients within a similar age range and found that permanent pacemaker implantation was required in only 1% to 5% of cases.

18. **Reviewer 3, comment 18:** The median time for long term mortality follow up needs to be described as this is very subjective in this age group

Authors' response to R3 comment 18: The parameters for long-term mortality vary. In the 28 papers selected, 19 listed follow-up times for long-term mortality ranging from 1 to 7.5 years, with a median of 3 years. As noted in the text, the lack of a uniform definition or method for assessing long-term mortality may create difficulties for anaesthetists, physicians, surgeons, and patients seeking to weigh the potential benefits of surgery versus optimal medical management of their cardiovascular disease.

19. **Reviewer 3, comment 19:** 383 and 317 report different median stays

Authors' response to R3 comment 19: Thank you for picking up this important error that we have made. As stated in our results section, fifteen publications reported the length of hospital stay, with a median of 12.5 days. Nine publications reported the length of ICU stay with a median of 7.1 days. This has been corrected in the discussion section. Thank you for pointing out this error.

20. **Reviewer 3, comment 20:** It was not clear what the outcome of the thematic analysis was. Ideally report this in a separate section.

Authors' response to R3 comment 20: Our statistical analysis plan initially included a thematic analysis of patient-reported outcomes and patient-reported measures to provide a comprehensive understanding of the patient experience following cardiac surgery in nonagenarians.

However, upon thorough review of the 27 included studies, we found that none reported PROMs or PREMs. This absence of patient-centered data precluded the planned thematic analysis. The lack of such outcomes in the current literature highlights a significant gap in our understanding of how nonagenarians perceive and evaluate their post-surgical quality of life, functional status, and overall satisfaction with cardiac surgical interventions. This finding underscores the need for future research to incorporate patient-reported outcomes in studies involving this unique and growing patient population.

21. **Reviewer 3, comment 21:** Table 3 - is there any way of summarising how many of the studies included reported this minimum dataset?

Authors' response to R3 comment 21: None of the studies included in this review included the full minimum dataset. For example, none of the studies included the standardised patient-centred outcomes listed at the bottom of the third column of Table 3. To emphasize this important point, we have mentioned this once again.

22. **Reviewer 3, comment 22:** The statistical method of weighting the different studies is not outlined. Please explain how studies contributed to the averages shown.

Authors' response to R3 comment 22: Thank you for this important question. Our scoping review intentionally omitted statistical weighting of included studies due to three key methodological considerations. First, the primary objective of scoping reviews is to map the breadth of evidence rather than synthesize quantitative outcomes, making statistical weighting methods like propensity scoring or raking inappropriate for this exploratory aim. Second, the inherent heterogeneity in study designs (including case series, observational cohorts, and small surgical registries), surgical approaches, and outcome reporting among nonagenarian cardiac surgery studies would render weighted comparisons statistically unreliable and clinically meaningless. Third, the limited pool of observational studies (n=27) with small sample sizes (many with 50% of studies reporting sample sizes of less than 30 patients) lacks the statistical power required for meaningful weighting adjustments.

Our focus on descriptive characterization of complications, mortality trends, and risk stratification patterns aligns with scoping review methodology that prioritizes comprehensive evidence mapping over quantitative synthesis. The decision reflects established guidance that weighting should be reserved for systematic reviews attempting meta-analyses of comparable outcome measures, particularly inappropriate here given the diversity in surgical populations and operative techniques across included studies.

23. **Reviewer 3, comment 23:** Overall, I found this study interesting but with several concerns about the precise methodology, as above. I am not an expert in scoping reviews, so have referred to the PRISMA guidance to assess this, but have the impression that this has been largely adhered to. Where there is discrepancy, I have sought to identify where improvements could be made. The relative paucity of experience with scoping reviews in the cardiac surgery literature is not a fault of the authors, but rather one of this reviewer. Nonetheless, the net effect is that the authors must tolerate a more didactic review

Authors' response to R3 comment 23: On the contrary, we are grateful for the care and effort that you put into this thoughtful review. We have responded to your questions as thoroughly as possible. Your comments have allowed us to improve the scientific rigor of the review, and we are appreciative for this constructive feedback.

Once again, our most sincere thanks to the Editor and the expert reviewers for dedicating their valuable time and expertise to review our manuscript. We deeply appreciate your thoughtful feedback and constructive suggestions, which have greatly contributed to improving the clarity and quality of our work. Your guidance has been instrumental in helping us refine our review, and we are grateful for the opportunity to benefit from your insights.

We hope that our manuscript is now suitable for publication in PLoS One. We look forward to hearing from you.

Sincerely,

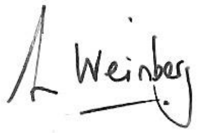

Professor Laurence Weinberg
BSc, MBBCh, MRCP, DipCritCareEcho, FANZCA, MD, PhD, ExecDipOL(Oxford)

Director, Department of Anaesthesia, Austin Hospital
Professor, Department of Critical Care, University of Melbourne, Australia
