# Supplementary material for: Outcomes and complications among nonagenarians undergoing cardiac surgery: A scoping review
Source: PLoS One. 2025 Sep 8;20(9):e0331755. doi: 10.1371/journal.pone.0331755 (PMC12416686; doi:10.1371/journal.pone.0331755)
Supplement: Attachment — Submitted filename: Response letter_PLOS_One_9July2025.pdf [file pone.0331755.s009.pdf]

Professor Alejandro Torrado Pacheco, Ph.D.
Associated Editor
PLOS One

Ref: Second Resubmission of PONE-D-24-26035

Title: Outcomes and complications in nonagenarians undergoing cardiac surgery: a scoping review

Dear Dr. Torrado Pacheco

Once again, my coauthors and I would like to thank you and the expert reviewers for taking the time to provide a very constructive and thoughtful review of our manuscript.

As requested, we have included the following items with our resubmission:

1. A point-by-point response letter (to follow) that addresses each issue raised by the Academic Editor and the Reviewers. I have uploaded this letter as a separate file labelled "Response to Reviewers."
2. A **marked-up copy** of the manuscript that **highlights in RED** changes made to the original version. This has been uploaded as a separate file labelled "Revised Manuscript with Tracked Changes."
3. An **unmarked version** of our revised paper without tracked changes. This has also been uploaded as a separate file labelled "Manuscript."

Comments from the Expert REVIEWERS

Reviewer 1: I appreciate the authors' thorough engagement with all feedback. The manuscript now appears well-prepared.

Authors' response: We sincerely appreciate the reviewer's recognition of this important research gap. We thank the Reviewer for their take taking to review the resubmission and for the constructive comment above.

Reviewer 4: Some methodological issues, highlighted in attached document, overall very interesting topic and merits publication with minor adjustments.

Authors' response: Thank you for taking the time to review our manuscript and for providing additional comment. We appreciate the opportunity to further revise our manuscript and enhance the scientific merits of the paper. Please find below a detailed response to each of your comments

1. Relevant topic, definite gap in the literature with regard to surgery on nonagenarians

Authors' response to Q1: We sincerely appreciate the reviewer's recognition of this important research gap. The paucity of evidence regarding surgical outcomes in nonagenarians represents a critical knowledge deficit in contemporary cardiac surgery practice. With the global nonagenarian population projected to exceed 30 million by 2030, and increasing prevalence of cardiovascular disease in this demographic, establishing evidence-based outcomes data is essential for informed clinical decision-making.

Our scoping review methodology was specifically chosen to systematically map the available evidence landscape, identify knowledge gaps, and provide a foundation for future research priorities in this rapidly expanding patient population.

2. Most points have been covered by the preceding 3 reviewers, I only have a couple of additions myself

Authors' response to Q2: We acknowledge the reviewer's collaborative approach and appreciate the opportunity to address additional methodological considerations that complement the feedback from previous reviewers. This iterative peer review process strengthens the scientific rigor and clinical relevance of our work.

3. Not sure why a pilot review was necessary, it seems like it was actually the initial step of the review and not a separate or discovery mission

Authors' response to Q3: Thank you for this important comment that we have discussed with our statistician who is also a co-author on this paper. In hindsight, we agree that the pilot review may not have been necessary. We were however unsure how many papers would need to be screened, and we felt that the pilot review was a standard methodological step to calibrate inter-rater consistency and ensure that inclusion/exclusion criteria were applied consistently before full screening.

We were also cognizant that the pilot review was implemented as a methodological quality assurance measure consistent with established scoping review guidelines. According to the JBI Manual for Evidence Synthesis and PRISMA-ScR recommendations, pilot testing serves multiple critical functions: (1) calibrating reviewer agreement and ensuring consistent application of inclusion/exclusion criteria, (2) validating data extraction instruments, and (3) identifying potential methodological challenges before full-scale implementation.

As described in the Methods, "Study selection and screening procedure", this step helped refine our screening tool, improve reviewer agreement, and ensured methodological transparency consistent with PRISMA-ScR and Levac et al.'s framework. Our pilot demonstrated substantial inter-rater reliability (Cohen's kappa = 0.643, 95.6% concordance), which provided methodological confidence for the complete review.

Whilst this approach is not always routine in scoping reviews it enabled a robust and systematic synthesis of the evidence and ensured reproducibility and reliability of findings.

4. I do agree with one of the reviews that there is some overlap between scoping and systematic reviews in this study, the study should, strictly speaking, report on the available literature, without seeking to synthesise it as that is the objective of a systematic review, as stated by the authors in line 147. for example, it is difficult to understand how they came up with the mortality rate of 10.5% when there is so much heterogeneity between the studies, with some studies having as small a sample of 9 and others as large a sample as over 2000, adding means from such differently powered studies may give an inaccurate result without weighting, which cannot be done for heterogenous studies.

Authors' response to Q4: We acknowledge the reviewer's important observation regarding methodological boundaries between scoping and systematic reviews. However, our approach aligns with established scoping review methodology as outlined in the PRISMA-ScR guidelines. Scoping reviews legitimately provide descriptive numerical summaries of available evidence without formal meta-analytical synthesis. The reported median mortality rate of 10.5% (IQR 7.2–14.6%) represents a descriptive statistic of the available evidence landscape, not a weighted pooled meta-analytical estimate. This has been added in to the Results sections (Lines 230-232)

We calculated medians and interquartile ranges precisely because of the heterogeneity you identify-this approach is less sensitive to extreme values and varying sample sizes than weighted means would be. The primary objective was to map the breadth of available evidence and identify patterns, which differs fundamentally from systematic review synthesis that would require formal quality assessment and meta-analytical techniques with appropriate weighting strategies.

Given this important comment, we have amended the Results and Discussion sections to more explicitly clarify that our findings are descriptive and derived from unweighted medians rather than means or pooled statistics, to avoid overinterpretation.

5. It is interesting to see the huge discrepancy in reported mortality rate in nonagenarians undergoing open heart surgery (10.5%) compared to nonagenarians undergoing non cardiac surgery (38%), It is difficult to believe that there is a reversal in the mortality rate in non-cardiac surgery compared to open cardiac surgery observed in the general population compared to that in such a high risk population. After all, a recent study in Italy identified the mortality in non-surgical nonagenarians to be 20.3% (Pancani, S., Lombardi, G., Sofi, F. *et al.* 12-month survival in nonagenarians inside the Mugello study: on the way to live a century. *BMC Geriatr* **22**, 194 (2022). https://doi.org/10.1186/s12877-022-02908-9). While the authors ascribe it to careful patient selection in cardiac surgery, could other factors have contributed, such as the statistical analysis problems mentioned above? Or if indeed this is a true result, could it be that this represents the findings in first-world countries, as most of the data was obtained from high-income countries?

Authors' response to Q5: The reviewer raises an astute observation about the apparent mortality paradox. We have included an additional paragraph in the discussion section that provided deeper insights. In the discussion section (Lines 357-369) we now state:

"Several factors beyond the statistical methods are likely to have contributed to this counterintuitive finding. First, the stringent patient selection for cardiac surgery in nonagenarians creates a highly selected cohort: patients must demonstrate adequate physiological reserves, cognitive function, and life expectancy to justify the procedural risk. In contrast, non-cardiac surgery populations might include emergent presentations with less rigorous preoperative optimization. Second, the availability of transcatheter alternatives might selectively direct higher-risk nonagenarians away from open surgery. Frail patients might be triaged toward transcatheter aortic valve replacement or medical management, thus further contributing to the lower observed mortality. Third, the reported increase in mortality of nonagenarians undergoing non-cardiac surgery represents a general nonagenarian population rather than a highly selected cardiac-surgery cohort. Fourth, the data reflect predominantly high-income countries with advanced perioperative care systems, specialized cardiac surgery centers, and comprehensive postoperative support.

In the limitations section (Lines 513-515), we have also added a cautionary note about generalizing these results to lower-resource settings where risk profiles and outcomes may differ significantly. We now state "*Finally, our findings cannot be generalized to lower-resource settings or lower- and middle-income countries, where risk profiles and outcomes may differ significantly."*

6. No mention as to whether the procedures documented were performed as elective or emergency procedures as the mortality rate may differ significantly between elective and emergency cardiac surgery

Authors' response to Q6: Thank you for raising this excellent point. We have reviewed each included paper once again to see if this was documented or captured in the original publication. Unfortunately, most included studies did not stratify outcomes based on surgical urgency.

We acknowledge this limitation (See lines 489-491), where we now state: "*Unfortunately, most included studies did not stratify outcomes by surgical urgency, thus constraining our ability to evaluate the differential effects of elective vs. emergent procedures."*

This omission constrains our ability to evaluate the differential impact of elective vs. emergent procedures. We have added this limitation to the Discussion as above, and updated Table 3 to

recommend that future studies report surgical urgency as a key preoperative variable for risk stratification.

7. Scoping review objectives differ between the introduction and the objectives, could do with some clarity, the third objective (Proms) was not met as there was insufficient data

Authors' response to Q7: Thank you for highlighting this discrepancy. We acknowledge the need for clarity between our introduction and formal objectives.

The apparent discrepancy reflects the evolution from our initial comprehensive research questions to the practical limitations encountered during evidence mapping. While we initially aimed to evaluate PROMs and PREMs comprehensively, the complete absence of such data in the literature necessitated reframing this as a critical gap identification rather than a quantitative analysis.

Both PREMS and PROMS represents legitimate scoping review outcomes-identifying what evidence exists and, equally importantly, what evidence is absent. We have clarified our objectives to reflect that PROMs/PREMs assessment was exploratory, with the understanding that absence of such data would itself constitute a significant finding. While PROMs and PREMs were included as objectives to identify literature gaps, we now explicitly state that no data were available, thus rendering that objective unmet. This reinforces the central finding of our review: a significant absence of patient-centered outcomes in this demographic.

We have revised the Introduction and Methods to ensure alignment with the final objectives stated.

- In the methods section (Lines 89-93) we state: "*Finally, as an exploratory outcome, the review identified whether patient-reported outcomes (PROMs) and experience measures (PREMs) have been included in studies examining outcomes in nonagenarians undergoing cardiac surgery, given that an absence of such data would itself constitute a notable finding.*"

- In the data synthesis and analysis section (Lines 211-213) we state: *"Our approach can therefore be accurately described as a narrative synthesis of quantitative findings, organized thematically around clinical domains (mortality, morbidity, and length of stay)."*

- In the section Patient-reported experience and outcomes measures section (Lines 320-321) we state: *"None of the included studies reported PROMs or PREMs; therefore, this study objective was unmet."*

8. According to the comments made in response to previous reviewer comments, no data could be found regarding patient satisfaction measures, yet it is mentioned in the objectives and methods under data extraction, and discussion see lines 92, 194, 195,204, 211,212 and 331 etc. Should this section then not be removed from the objectives and methods, and a comment made only in the discussion, identifying it as a gap in the literature?

Authors' response to Q8: The reviewer correctly identifies an apparent inconsistency in our methodology. This has been corrected, and this section has been removed from the objectives and methods.

- The methods section (Lines 89-93) states: "*Finally, as an exploratory outcome, the review identified whether patient-reported outcomes (PROMs) and experience measures (PREMs) have been included in studies examining outcomes in nonagenarians*

> *undergoing cardiac surgery, given that an absence of such data would itself constitute a notable finding."*

The methodology sections appropriately described our comprehensive search strategy, while the results and discussion sections report the significant finding that no such data exists. This absence of patient-centered outcome measures represents a critical knowledge gap that our review identified.

9. Again in the data analysis section we find some overlap in terms of methodology, thematic analysis is a qualitative analytic tool and depends on the conceptual framework and selection of appropriate paradigm, the context of which is of the utmost importance, this makes it technically difficult to code and draw themes from an analysis of retrospective quantitative data obtained from thousands of patients who had different research tools used in many varying countries with differing languages, especially as no patient satisfaction measures were identified in the data, the appropriate analysis of such data in the case of a scoping review is simply narrative

Authors' response to Q9: The reviewer raises important considerations about thematic analysis methodology. We acknowledge that our use of the term "thematic analysis" may be imprecise in this context. Given the absence of qualitative data or patient-reported outcomes in the included studies, formal thematic analysis was indeed impossible. Our approach is more accurately described as narrative synthesis of quantitative findings, organized thematically around clinical domains (mortality, morbidity, length of stay).

We have revised our methodology description to more accurately reflect this narrative approach rather than formal thematic analysis, which requires qualitative data and interpretive frameworks as the reviewer correctly notes. Given the paucity of such data, we have removed thematic analysis from our stated analytical methods and revised the Data Analysis section to reflect a narrative synthesis alone. These changes help ensure methodological consistency and prevent overreach.

Thank you for this important suggestion.

10. According to the authors, the majority of the articles, 20 to be specific, were obtained from grey literature and reference list search, can they specify the sources? even in the Prisma flow diagram, the sources are only described as grey literature. While only 9 came from the database search, is there a reason why so few databases were included in the search? In essence, only two databases returned results since no RCTs (from the 5 identified from CCRCT) formed part of the results

Authors' response to Q10: We acknowledge the reviewer's request for enhanced transparency regarding our grey literature sources. Upon careful review of Figure 1, we recognize that the diagram is misleading and have accordingly revised it to accurately reflect our search criteria.

The 20 additional articles were identified through systematic hand-searching of reference lists from included studies and targeted searches of relevant professional organization websites, conference proceedings, and institutional repositories. All of these articles were subsequently excluded as they did not meet our specified patient population criteria. This multi-source approach aligns with established comprehensive scoping review methodology.

In the revised manuscript we now state: "*From the initial screening process, a total of 1826 articles were identified. An additional 20 articles were identified through a search of the grey literature and reference tracking, to supplement the primary database search. However, no eligible articles were identified from the grey literature. In total, 81 full-text publications were assessed for eligibility, among which 28 studies [17–44] met the inclusion criteria and were included in the final analysis. The PRISMA flow diagram detailing the selection of studies is*

presented in Fig 1. Although a narrative synthesis approach was used for quantitative data, no qualitative themes were identified. In addition, our findings are descriptive and were derived from unweighted medians rather than means or pooled statistics, to avoid overinterpretation.

The revised figure is provided below. We appreciate the reviewer's feedback, which has enhanced the clarity of our methodology.

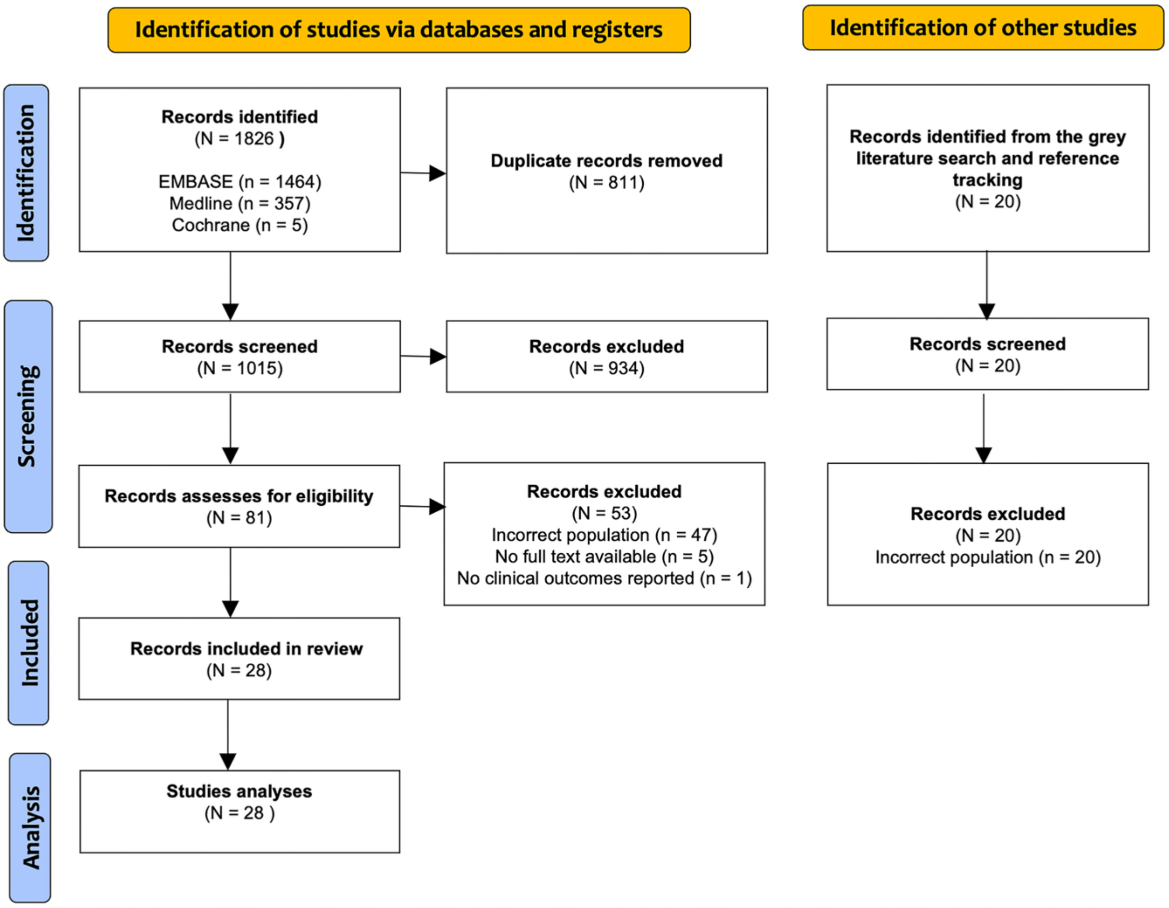

11. There is a mention of the impact of the findings on healthcare resource allocation in the introduction (line 82 & 83), yet in the exclusion factors, papers discussing the economic impact were excluded, this I think is one of the most highly relevant discussion points, especially for low to middle income countries that use morbidity and mortality research such as this to guide decision making, since essentially, this paper is about the clinical factors informing the ethics impacting the decision to undergo high risk surgery in a high risk group.

    Indulge me to play devils advocate but while every life is precious, an argument for ageism to a certain extent may have to be tolerated, one cannot ignore the fact that increased length of stays and morbidity impact economically on the healthcare system, and due to the increased comorbidities in this age group as correctly identified by the authors, including the normal physiological changes of ageing. Indeed, once a surgical treatment course is embarked upon, is it not less ethical to terminate it in poor responders than not to have begun at all? can an ethical justification be made for exposing high risk individuals who cannot contribute to fiscal income, and may not benefit in terms of appreciable prolongation of life (with less than half of patients surviving 5 years), and in all likelihood, may experience a poorer post operative quality of life (again, data in the article reports up to a quarter of patients suffering a loss of

functional capacity, considering of course whether such quality did indeed exist at baseline in the case of one with dementia or other cognitive diseases), who also might increase the economic burden on the healthcare system?

Authors' response to Q11: This is a profoundly important point. We excluded studies focusing solely on economic evaluations to maintain clinical outcome relevance. We aimed to focus the scope strictly on "clinical" metrics and outcomes to maintain review coherence.

In the revised manuscript, we now explicitly acknowledge this limitation and its implications for healthcare policy, particularly in low- and middle-income countries. This ethical dimension is further discussed in the Discussion section, including arguments around opportunity costs, age-related equity, and the moral imperative for individualized decision-making in high-risk populations.

In the new subsection in the Discussion, we reflect on the ethical tensions between individual benefit, quality of life, and resource allocation-especially in resource-constrained environments. We have cited recent literature discussing ethical frameworks for surgical decisions in the elderly and added commentary on how age-related health economics may shape future triage decisions.

In the Discussion section of the revised manuscript (Lines 517-543), we now state:

"Ethical tensions, individual benefits, and resource allocation
Providing open cardiac surgery to nonagenarians presents complex health economic and ethical challenges, particularly in resource-constrained settings and lower- and middle-income countries, where healthcare systems often operate with limited capacity and funding. The cost of cardiac surgery is substantial, and prolonged ICU stays, increased complication rates, and extended hospital admissions are often involved, thereby contributing to higher healthcare expenditures than required for younger cohorts. Moreover, in lower- and middle-income countries, where access to expensive interventions must be weighed against broader public health needs, questions arise regarding whether prioritizing high-cost procedures in the oldest populations constitutes an equitable or sustainable use of limited resources. Consequently, tension exists between the ethical principle of individual beneficence, providing the best possible care to a single patient, and the utilitarian imperative to maximize health outcomes at the population level. Ivashkov and Van Norman have argued that surgical decision-making in older adults must balance respect for autonomy with realistic expectations of benefit and resource stewardship, particularly when quality of life might be diminished by frailty, comorbidities, or cognitive impairment [71]. Similarly, Altawalbeh et al. have highlighted how age-related ethical frameworks must incorporate considerations of distributive justice, given that the opportunity cost of providing intensive treatment to nonagenarians might result in denying care to younger or more functionally independent patients [46]. Because surgical outcomes in the oldest populations remain highly variable, and data on long-term functional recovery remain sparse, healthcare policymakers must increasingly confront the moral dilemma of whether aggressive interventions are justifiable when life expectancy and quality-adjusted life years might be limited. Accordingly, transparent, culturally sensitive, and evidence-informed triage frameworks are essential to support ethical and economically sound decision-making in cardiac surgery for the oldest populations. As demographics shift globally, policies must reconcile economic realism with ethical imperatives to avoid discriminatory rationing, and must uphold the "fair innings" principle aimed at equitable health opportunities across lifespans [72,73]."

Additional References addedin to the main manuscript

1. Ivashkov Y, Van Norman GA. Informed consent and the ethical management of the older patient. Anesthesiology Clinics. 2009;27(3):569–580. https://doi.org/10.1016/j.anclin.2009.07.016
2. Altawalbeh SM, Alkhateeb FM, Attarabeen OF. Ethical issues in consenting older adults: academic researchers and community perspectives. Journal of Pharmaceutical Health Services Research. 2019;11:25–32. https://doi.org/10.1111/jphs.12327
3. Oehring D, Gunasekera P. Ethical Frameworks and Global Health: A Narrative Review of the "Leave No One Behind" Principle. Inquiry. 2024 Jan-Dec;61:469580241288346. doi: 10.1177/00469580241288346. PMID: 39385394; PMCID: PMC11465308.

Thank you for this excellent insight and suggestion.

12. Overall I enjoyed reading this study, I think it is highly relevant as someone who practices geriatric cardiac for non-cardiac anaesthesia, I suspect however that the findings are rather optimistic compared to my experience but that may be influenced by the fact that I live in a third world county.

Authors' response to Q12. We are grateful for your insightful reflection. We recognize that the findings in our review, drawn largely from high-income countries and retrospective data, may appear more optimistic than clinical experiences in low-resource settings. We hope that we have now addressed this issue in the Discussion section and added cautionary language to avoid overgeneralizing results to settings with differing health infrastructure or patient demographics.

On behalf of my co-authors, I thank you for your excellent comments.

Other Minor Revisions:

1. We have meticulously adhered to the journal's Instructions for Authors and have made minor adjustments to grammar and syntax to further improve the clarity and readability of the manuscript.
2. American English spelling has been used consistently throughout the manuscript.
3. The manuscript has been thoroughly revised to ensure that it is written entirely in the third person.

Once again, our most sincere thanks to the Editor and the expert reviewers for dedicating their valuable time and expertise to review our manuscript. We deeply appreciate your thoughtful feedback and constructive suggestions, which have greatly contributed to improving the clarity and quality of our work. Your guidance has been instrumental in helping us refine our review, and we are grateful for the opportunity to benefit from your insights.

We hope that our manuscript is now suitable for publication in PLoS One. We look forward to hearing from you.

Sincerely,

L Weinberg

Professor Laurence Weinberg
BSc, MBBCh, MRCP, DipCritCareEcho, FANZCA, MD, PhD, ExecDipOL(Oxford)

Director, Department of Anaesthesia, Austin Hospital
Professor, Department of Critical Care, University of Melbourne, Australia
