# Supplementary material for: Outcomes and complications among nonagenarians undergoing cardiac surgery: A scoping review
Source: PLoS One. 2025 Sep 8;20(9):e0331755. doi: 10.1371/journal.pone.0331755 (PMC12416686; doi:10.1371/journal.pone.0331755)
Supplement: Attachment — Submitted filename: scoping review open heart surgery on nonagenarians.docx [file pone.0331755.s007.docx]

scoping review on the complications of open heart surgery on nonagenarians.

Comments

1. Relevant topic, definite gap in the literature with regard to surgery on nonagenarians
2. Most points have been covered by the preceding 3 reviewers, I only have a couple of additions myself
3. Not sure why a pilot review was necessary, it seems like it was actually the initial step of the review and not a separate or discovery mission
4. I do agree with one of the reviews that there is some overlap between scoping and systematic reviews in this study, the study should, strictly speaking, report on the available literature, without seeking to synthesise it as that is the objective of a systematic review, as stated by the authors in line 147. for example, it is difficult to understand how they came up with the mortality rate of 10.5% when there is so much heterogeneity between the studies, with some studies having as small a sample of 9 and others as large a sample as over 2000, adding means from such differently powered studies may give an inaccurate result without weighting, which cannot be done for heterogenous studies.
5. It is interesting to see the huge discrepancy in reported mortality rate in nonagenarians undergoing open heart surgery (10.5%) compared to nonagenarians undergoing non cardiac surgery (38%), It is difficult to believe that there is a reversal in the mortality rate in non-cardiac surgery compared to open cardiac surgery observed in the general population compared to that in such a high risk population. After all, a recent study in Italy identified the mortality in non-surgical nonagenarians to be 20.3% (Pancani, S., Lombardi, G., Sofi, F. *et al.* 12-month survival in nonagenarians inside the Mugello study: on the way to live a century. *BMC Geriatr* **22**, 194 (2022). <https://doi.org/10.1186/s12877-022-02908-9>). While the authors ascribe it to careful patient selection in cardiac surgery, could other factors have contributed, such as the statistical analysis problems mentioned above? Or if indeed this is a true result, could it be that this represents the findings in first-world countries, as most of the data was obtained from high-income countries?
6. No mention as to whether the procedures documented were performed as elective or emergency procedures as the mortality rate may differ significantly between elective and emergency cardiac surgery
7. Scoping review objectives differ between the introduction and the objectives, could do with some clarity, the third objective (Proms) was not met as there was insufficient data
8. According to the comments made in response to previous reviewer comments, no data could be found regarding patient satisfaction measures, yet it is mentioned in the objectives and methods under data extraction, and discussion see lines 92, 194, 195,204, 211,212 and 331 etc. Should this section then not be removed from the objectives and methods, and a comment made only in the discussion, identifying it as a gap in the literature?
9. Again in the data analysis section we find some overlap in terms of methodology, thematic analysis is a qualitative analytic tool and depends on the conceptual framework and selection of appropriate paradigm, the context of which is of the utmost importance, this makes it technically difficult to code and draw themes from an analysis of retrospective quantitative data obtained from thousands of patients who had different research tools used in many varying countries with differing languages, especially as no patient satisfaction measures were identified in the data, the appropriate analysis of such data in the case of a scoping review is simply narrative
10. According to the authors, the majority of the articles, 20 to be specific, were obtained from grey literature and reference list search, can they specify the sources? even in the Prisma flow diagram, the sources are only described as grey literature. While only 9 came from the database search, is there a reason why so few databases were included in the search? In essence, only two databases returned results since no RCTs (from the 5 identified from CCRCT) formed part of the results
11. There is a mention of the impact of the findings on healthcare resource allocation in the introduction (line 82 & 83), yet in the exclusion factors, papers discussing the economic impact were excluded, this I think is one of the most highly relevant discussion points, especially for low to middle income countries that use morbidity and mortality research such as this to guide decision making, since essentially, this paper is about the clinical factors informing the ethics impacting the decision to undergo high risk surgery in a high risk group. Indulge me to play devils advocate but while every life is precious, an argument for ageism to a certain extent may have to be tolerated, one cannot ignore the fact that increased length of stays and morbidity impact economically on the healthcare system, and due to the increased comorbidities in this age group as correctly identified by the authors, including the normal physiological changes of ageing. Indeed, once a surgical treatment course is embarked upon, is it not less ethical to terminate it in poor responders than not to have begun at all? can an ethical justification be made for exposing high risk individuals who cannot contribute to fiscal income, and may not benefit in terms of appreciable prolongation of life ( with less than half of patients surviving 5 years), and in all likelihood, may experience a poorer post operative quality of life (again, data in the article reports up to a quarter of patients suffering a loss of functional capacity, considering of course whether such quality did indeed exist at baseline in the case of one with dementia or other cognitive diseases), who also might increase the economic burden on the healthcare system?
12. Overall I enjoyed reading this study, I think it is highly relevant as someone who practices geriatric cardiac for non-cardiac anaesthesia, I suspect however that the findings are rather optimistic compared to my experience but that may be influenced by the fact that I live in a third world county.
